# Antibodies raised against a Sunn bug (*Eurygaster integriceps* Put.) recombinant protease, rGHP3p2, can inhibit gluten‐hydrolyzing activity

**DOI:** 10.1002/fsn3.1361

**Published:** 2019-12-30

**Authors:** Vyacheslav Dolgikh, Alexander Tsarev, Sergey Timofeev, Vladimir Zhuravlyov, Igor Senderskiy, Alison Lovegrove, Alexander Konarev

**Affiliations:** ^1^ Department of Molecular Plant Protection All‐Russian Research Institute of Plant Protection (VIZR) Pushkin, St. Petersburg Russia; ^2^ Plant Biology and Crop Science Department Rothamsted Research Harpenden UK; ^3^ Department of Agicultural Entomology All‐Russian Research Institute of Plant Protection (VIZR) Pushkin, St. Petersburg Russia

**Keywords:** active center, antibody, protease, protease inhibitory activity, Sunn bug, wheat gluten

## Abstract

Sunn pest or Sunn bug, *Eurygaster integriceps* Put., salivary gland proteases are responsible for the deterioration of wheat flour quality during dough mixing, resulting from gluten hydrolysis. These proteases are highly heterogeneous and show low sensitivity to most types of proteinaceous inhibitors, meaning that such inhibitors cannot be used to prevent gluten damage. The present study describes the generation of a specific peptide antibody, raised against the active center of the recombinant gluten‐hydrolyzing protease (GHP3). The recombinant protein, encoding two repeats of the GHP3 sequence element involved in forming the S4 pocket and binding of substrate at position P4, was designed and expressed in *Escherichia coli*. The antibodies raised to this recombinant protein showed inhibitory activity against the GHP3 protease. The results indicate that it is possible to design specific antibodies to inhibit wheat‐bug gluten‐hydrolyzing proteases.

## Graphical abstract

Sunn pest or Sunn bug salivary gland proteases are responsible for the deterioration of wheat flour quality during dough mixing, resulting from gluten hydrolysis. The present study describes the generation of a specific peptide antibody, raised against the active center of the recombinant gluten‐hydrolyzing protease (GHP3). The antibodies raised to this recombinant protein showed inhibitory activity against the GHP3 protease that indicates the possibility to design specific antibodies to inhibit wheat‐bug gluten‐hydrolyzing proteases.

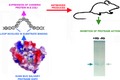

## 1 INTRODUCTION

Proteases are involved in many processes occurring in living organisms including, normal digestion, protein processing, and blood clotting but they are also involved in damaging pathologies such as infection, inflammation, and thrombosis. Limiting undesirable proteolytic activity is important not only in medicine (Farady & Craik, 2010), but also in agriculture, particularly in relation to plant resistance to pests and pathogens (Hamza et al., 2018; Islamov, Kustova, & Ilin, 2012; Konarev, 2017; Rasoolizadeh et al., 2016). Proteases are required for the assimilation of plant proteins by insects, microorganisms, and other phytophages, which results in a decrease in crop yield and quality. Sunn pest (or Sunn bug), *Eurygaster integriceps* Put. (Hemiptera: Heteroptera: Scutelleridae), salivary gland proteases that remain in damaged wheat grains after insect feeding are able to exert their activity during dough mixing. The Sunn pest proteases hydrolyze the main gluten proteins, glutenins and gliadins, significantly impairing the technological qualities of the dough and the products made from it, for example, bread and pasta (Allameh, Kadivar, & Shahedi, 2015; Dizlek & Ozer 2016; Sivri, Sapirstein, Köksel, & Bushuk, 1999). Many of the known methods to protect or partially restore gluten quality from damaged grain are based on the usage of various reagents or technologies, such as chemical oxidants, which are often not effective or safe for human use (Wolf et al., 1998). Just as drugs are developed in medicine to suppress the destructive activity of proteases based on proteinaceous inhibitors from plants and animals (Gitlin‐Domagalska et al., 2017; Malik et al., 2015), a similar approach could be used to protect wheat grain proteins from damage by Sunn pest proteases. The application of this approach is complicated in the case of Sunn pest proteases by the high heterogeneity of salivary gland proteases and the low sensitivity of these proteases to the main types of known protease inhibitors (Konarev et al., 2011, 2019). Despite the fact that proteinaceous protease inhibitors are extremely diverse in size and amino acid sequences, their activity is carried out through only a few general mechanisms of action (Krowarsch, Cierpicki, Jelen, & Otlewski, 2003; Laskowski & Kato, 1980). One of the most common inhibitory mechanisms, competitive inhibition, is based on the inhibitor substituting for the natural substrate in the active site of the protease. In contrast to the substrate, the inhibitor, contacting the active site of the enzyme, forms a stable complex with the latter, which prevents it from carrying out enzymatic activity, as access of the substrate to the active center of the protease is blocked. A second inhibitory mechanism, allosteric inhibition, occurs when the inhibitor binds to the enzyme outside of the active site, but the binding results in a conformational change such that the active site is no longer available for substrate binding. These mechanisms are often interrelated and individual “two‐headed” inhibitors can use both mechanisms in parallel (Farady & Craik, 2010). Such inhibitors with the required specificity can be constructed using, for example, computer simulation methods or phage display (Scott & Taggart, 2010; Stoop & Craik, 2003). The disadvantage of the use of peptide inhibitors is that there is a high degree of conservation of the structures at the active centers of enzymes, which can therefore result in inhibitors with a broad range of inhibitory activities. (Schneider et al., 2012). For the suppression of specific proteases, it is of interest to use antibodies as inhibitors (Conrad & Floss, 2010; Sgier, Zuberbuehler, Pfaffen, & Neri, 2010). Amino acid sequences of enzymes and secondary and tertiary structures are extremely diverse. Antibodies raised against these diverse polypeptides are therefore likely to be highly specific. The object of the described work was to determine whether it was possible to produce an antibody able to specifically inhibit the activity of one of the proteases synthesized in the Sunn pest salivary glands, GHP3. A recombinant polypeptide was produced based on the specific S4 pocket at the active center in GHP3 and a polyclonal antibody raised against this. Inhibitory activity of the antibody was tested against the recombinant form of Sunn bug protease, rGHP3p2.

## 2 MATERIALS AND METHODS

### 2.1 Comparison of Sunn pest proteases with those of other organisms

Comparison of the amino acid sequences that are part of the active sites of the Sunn pest proteases (ADP06392, ADP06390, and ADP06391) and other organisms was performed using the Blast algorithm (http://blast.ncbi.nlm.nih.gov/).

### 2.2 DNA construct and heterologous expression of chimeric protein in *E. coli*

DNA fragment encoding Val120‐Pro153 peptide (VPVASWIEHEQYYGPINDAGRTINDIALLMLAKP) of GHP3 was PCR amplified using GTGTggatccGTACCAGTCGCTAGTTGGATCGAG (forward) and ACCCagatctAGGTTTGGCCAACATCAGCAGG (reverse) pair of primers (BamHI and BglII sites are underlined), DreamTaq Green PCR Master Mix (Thermo Fisher Scientific), and cDNA of *E. integriceps* GHP3 previously cloned in pRSET plasmid (Dolgikh, Senderskii, & Konarev, 2014). PCR product of about 110 bp was gel‐purified, digested with BamHI/BglII, ligated using T4 DNA ligase, and redigested with the same enzymes to eliminate conjunctions of BamHI/BglII ends. The pool of DNA fragments encoding oligomers of Val120‐Pro153 peptide were ligated into pRSETa vector after linearizing with BamHI/BglII enzymes, followed by dephosphorylation of the ends. *E. coli* XL‐1 Blue MRF' cells were transformed with ligation products via electroporation at 1,700 V using Electroporator 2510 (Eppendorf). Bacterial colonies on LB plates containing 0.15 mg/ml ampicillin were analyzed by PCR using the above reverse and T7 forward primers. Plasmid DNA from a single bacterial colony carrying an insert of about 450 bp in the correct sense orientation was transformed into *E. coli* BL21 (DE3)‐derived C41 cells (Miroux & Walker, 1996) by electroporation, and fresh colonies selected on LB plates with ampicillin were inoculated in 25 ml of the same liquid broth. The cultures were grown to an OD of 0.4 at 600 nm, and expression was induced by the addition of 0.7 mM IPTG (final concentration) followed by incubation for 15 hr at 37°C. After culturing, the bacterial cells were pelleted at 3,000 *g* for 15 min and sonicated in 1 ml of 50 mM Tris‐Cl (TB, pH 7.5). The inclusion bodies (IBs) were spun down at 1,500 *g* for 10 min, carefully washed with TB and dissolved in 8 M urea followed by removal of insoluble debris at 14,000 *g* for 5 min.

### 2.3 Production and purification of polyclonal antibodies

The recombinant protein solubilized in 8 M urea was diluted tenfold with PBS (50 mM sodium phosphate, 0.15 M NaCl [pH 7.5]), mixed with an equal volume of Freund's adjuvant (Sigma; complete for first injection, incomplete for those following) and used for immunization. Mice were immunized by four intraperitoneal injections (0.08 mg protein per injection) at 10‐day intervals.

Ten days after the last immunization, 0.5 ml of blood was collected and sera were analyzed by immunoblotting. To purify specific antibodies, about 0.1 mg of recombinant protein was separated by SDS–polyacrylamide gel electrophoresis (PAGE) on a 12% gel, transferred on nitrocellulose membrane and stained with Ponceau S. Strips of membrane corresponding to the recombinant protein were precisely cut, blocked for 1 hr in TTBS (50 mM Tris‐HCl pH 7.4, 150 mM NaCl, 0.05% Tween‐20) with 1% bovine serum albumin (BSA), and incubated with 1.5 ml immune serum diluted 1:5 in TTBS for 12 hr at 4°C. Nitrocellulose strips were washed with TTBS, then with TBS (TTBS without Tween‐20) and incubated with 400 μl 0.2 M glycine‐HCl (pH 2.5) to elute antibodies. Aspirated solution was immediately neutralized by the addition of 45 μl 1 M Tris (untitrated) and kept on ice. After addition of 5 μl of 5 M, NaCl antibodies were concentrated using Microcon Centrifugal Filter Units (Millipore) and used for inhibitor activity assays. As a control, antibodies were removed from this solution by ultrafiltration.

### 2.4 Production of recombinant protease and analysis of its activity

The active recombinant form of the rGHP3p2 protease was obtained by heterologous expression of the previously cloned cDNA sequence (HM579787.14; Konarev et al., 2011) in the *Pichia pastoris* yeast cells (Dolgikh et al., 2014). The activity of the protease was assessed by the hydrolysis of peptide substrates R1N5 and R3N3, recombinant homologues of repeating sequences of glutenin hexa‐ and nonapeptides with molecular mass near 21.5 kDa (Wellner et al., 2006), kindly provided by Dr. J. Marsh. Peptides contain repeats PGQGQQ/GYYPTSLQQ and PGQGQP/GYYPTSLQQ in different combinations. These substrate peptides were selected because they are soluble in 0.05 M Tris‐HCl buffer pH 8.5, produce a single band following electrophoresis, and are highly sensitive to hydrolysis. Protease assay mix was made up of 2 μl of recombinant protease in 0.05 M Tris‐HCl pH 8.5 buffer and 2 μl of antibody or control solution in the same buffer. After 15 min, 2 μl of peptide substrate (4 μg/μl) was added and after mixing the assay mixture incubated for 6 hr at 40°C in tightly closed tubes. At the end of the assay, the reaction was stopped by the addition of an equal volume of SDS sample buffer which consisted of 2% SDS‐Na, 0.2 M dithioerythritol (DTE), 10% glycerol, and 50 mM Tris‐HCl (pH 6.8). The assay mix was then incubated at 98°C for 3 min, cooled, and applied to a 12.5% polyacrylamide gel for electrophoresis (Laemmli, 1970). Proteins were stained with Coomassie G‐250. Experiments on analysis of inhibitory activity of antibodies were repeated four times with two different peptide substrates.

## 3 RESULTS AND DISCUSSION

### 3.1 Rationale for selection of oligopeptide sequence used to produce recombinant protein against which antibodies were raised

Previous work had highlighted that several sites participate in the formation of the active site of the enzyme (GHP3; Konarev et al., 2011). In the present study, the GPINDAG sequence fragment (residue Gly 85‐Gly91, based on the mature protein; or Gly133‐Gly139, based on the full‐length sequence) which forms a loop corresponding to the S4 pocket of the active center of GHP3 (Table 1) was selected for recombinant chimeric expression. The glutamine residue at position P4 relative to the site of hydrolysis of the wheat glutenin hexa/nonapeptide element (PG**Q**GQQ/GYYPTSLQQ) fits into this pocket, as observed in molecular models (Konarev et al., 2011). The loop is involved in substrate binding and in molecular models is at the surface of the enzyme molecule. This suggested that it would be a good target for the production of specific antibodies, which might have the potential to act as specific inhibitors of the protease GHP3. To ensure specificity of inhibitor action, that is, that the inhibitory activity would only be against the Sunn pest protease and not beneficial proteases present in nontarget organisms, the selected oligopeptide sequence was compared with digestive protease sequences from a range of species. All enzymes listed in Table 1 are trypsin‐like and belong to one of the most extensive subfamilies of the animal extracellular peptidases, S1A (Page & Di Cera, 2008). Sequence comparison of proteases from *E. integriceps* adults salivary glands from different regions of Russia (APD0639(2)/(1)/(0)), chymotrypsin‐like protease 1 of *Halyomorpha halys* (a pest of many crops), closely related to the Scutelleridae family Pentatomidae (XP_014274701), and digestive proteases from more distantly related pests, crustaceans, and mammals are all shorter than the oligopeptide selected for recombinant protein production and show little homology (Table 1). Trypsin, from bovine pancreas and the most important human digestive serine proteases, pancreatic trypsin, chymotrypsin, and elastase, do not contain the GPINDAG fragment of GHP3. Additionally, the gut enterokinase AHC28777 is significantly different in amino acid sequence from GHP3. Therefore, the antibodies produced, with inhibitory activity toward the Sunn bug protease, are unlikely to have activity against mammalian endogenous proteases.

**Table 1:** Sequence alignment of oligopeptide (Ser115‐Ala158) from GHP3 protease (ADP06392) with those of other digestive or related to digestion proteases from Heteroptera insects, crustacean, and mammals

Species	Enzyme	Organ	Accession	1st Res. nos.	
*Eurygaster integriceps* Put	GHP3	sg	ADP06392	115	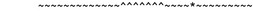 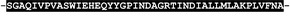
*E. integriceps*	GHP1	sg	ADP06390	115	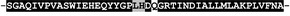
*E. integriceps*	GHP2	sg	ADP06391	115	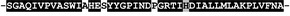
*Halyomorpha halys* Stal	Chymotrypsin	–	XP_014274701	111	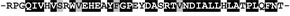
*H. halys*	Chymotrypsin	–	XP_014274066	83	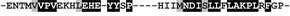
*Lygus lineolaris *(Palisot)	Trypsin	sg	AHY81279	123	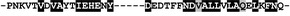
*L. distinctifemur* Menke	Venom protease	sg	ATU82413	130	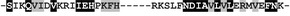
*Creontiades dilutus* Stal	Chymotrypsin	sg	AAL15154	115	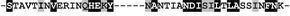
*Astacus leptodactylus* Eschscholtz	Trypsin	hp	2F91_A	73	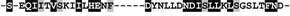
*Bos taurus* L.	Trypsin	p	NP_001107199	86	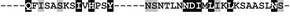
*Homo sapiens* L.	Elastase	p	AAA52380	101	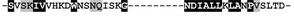
*H. sapiens*	Enterokinase	gut	AHC28777	66	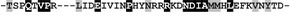
*H. sapiens*	Trypsin	p	NP_002760	84	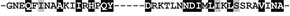
*H. sapiens*	Chymotrypsin	p	CAA74031	81	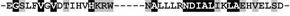

Note

Residues identical to those of the GHP3 Ser115‐Ala158 oligopeptide are shown in black boxes; conserved (positive) substitutions are in gray boxes; nonrelated residues are in black letters in white boxes. ~~~, selected oligopeptide sequence inserted in recombinant protein used for immunization of mice. ^^^^^^^, fragment of sequence forming loop involved in S4 pocket of GHP3 active center. *, catalytic aspartate residue.

Abbreviations: hp, hepatopancreas; p, pancreas; Res. nos., numbers of first residues of sequences shown in table; sg, salivary glands.

### 3.2 Antigen design

**Figure 1:**
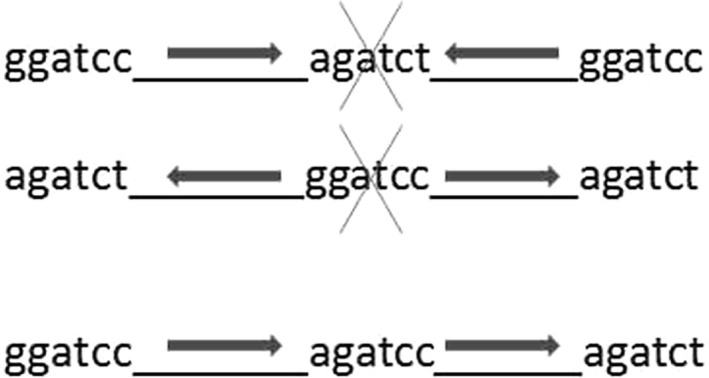
Preparation of DNA fragments encoding oligomers of the peptide Val120‐P153

Since small peptides are not immunogenic, we amplified a DNA fragment encoding a 34mer amino acid peptide containing the motif GPINDAG along with flanking sequences. This sequence was ligated, in the correct sense orientation with the target oligopeptide using BamHI and BglII (Figure 1). Since BamHI and BglII restrictases are isoschizomers, simultaneous treatment of the obtained PCR products with both enzymes, and ligation in the presence of T4 DNA ligases after repeated treatment with BamHI/BglII resulted in correctly oriented DNA fragments joined in one chain.

**Figure 2:**
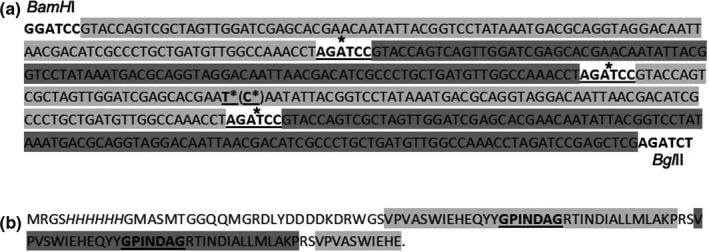
Production of recombinant protein carrying copies of the GHP3 oligopeptide Val120‐Pro153. (a) The nucleotide sequence cloned into the pRSETa vector and encoding 4 copies of the GHP3 oligopeptide Val120‐Pro153. * mark the internal missing restriction sites and the replacement of nucleotide C with T. (b) Amino acid sequence of the recombinant protein containing two copies of the GPINDAG motif (underlined) with flanking sequences in the Val120‐Pro153 oligopeptide (light gray background). The N‐terminal polyhistidine sequence is marked in italics

Despite the fact that the pRSET vector was used for cloning, a fragment of about 450 bp (which equates to four copies of the PCR product) was produced. However, the size of the recombinant protein was approximately 13 kDa instead of the expected 21 kDa. Sequence analysis of the construct confirmed the insertion of four copies of the Val120‐Pro153 peptide into the expression vector. However, PCR cloning had also led to a premature stop codon in the cloned sequence due to the replacement of nucleotide C with T, marked * (Figure 2a). This resulted in the shortened form of the recombinant protein, with size ~13.3 kDa and carrying only two copies of the GPINDAG motif with flanking sequences (Figure 2b).

**Figure 3:**
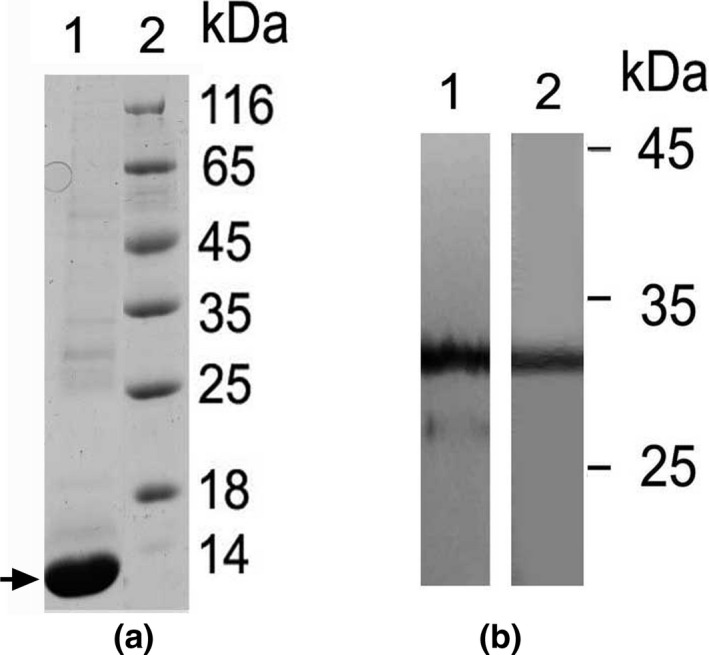
Analysis of the recombinant chimeric protein containing two copies of the oligopeptide Val120‐Pro153 and the antibodies to it. (a) SDS‐PAGE of recombinant protein (1, arrowed) and molecular weight markers (2). (b) Immunoblotting of proteins of the Sunn pest salivary glands with use of immune serum to chimeric protein (1) and fraction of antibodies purified by affinity chromatography on immobilized chimeric protein (2)

However, since the ~13 kDa recombinant protein was very efficiently produced in bacteria (about 0.2 g per liter of culture) and easily isolated, in very pure form (Figure 3a, track 1) from the insoluble protein inclusion bodies, this chimeric protein was used for mouse immunization. Antibodies were isolated from sera by affinity chromatography as described in materials and methods.

Immunoblots of Sunn pest salivary gland proteins, probed with the antibodies produced showed specific recognition of a protein band of about 31 kDa, which corresponds to the molecular weight of the protease GHP3 in zymogen form (Konarev et al., 2019; Figures 3b, 1).

Antibodies were purified from the immunoblots, as described in M and M. Track 2 (Figure 3b) confirms the presence of antibodies in fraction eluted from blotted polypeptide and used for analysis of inhibitory activity.

### 3.3 Detection of protease inhibitory activity of antibodies

**Figure 4:**
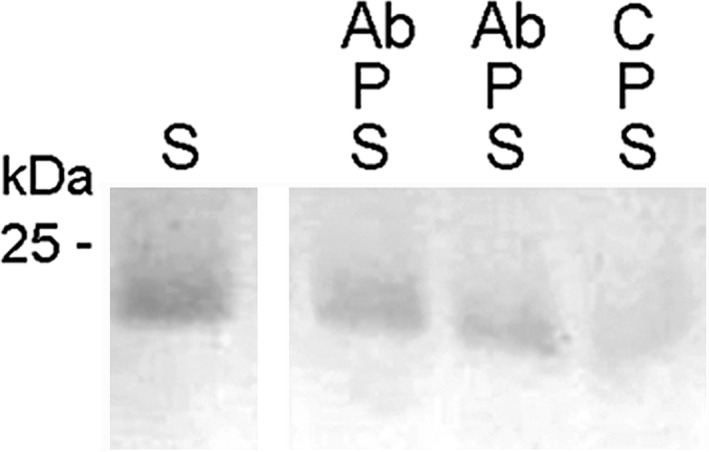
Protease activity assay with substrate peptide R1N5. S, substrate alone; P, recombinant protease rGHP3p2; Ab, antibody purified by affinity chromatography; C, control solution (no antibody) separated from the purified antibodies by ultrafiltration at their concentration

Recombinant peptide substrates R1N5 or R3N3 were used to test for inhibitory activity of antibodies toward recombinant GHP3 protease (Figure 4). Electropherogram analysis showed that antibodies inhibit protease activity against glutenin substrate.

## 4 CONCLUSION

Antibodies produced to the chimeric recombinant protein, specific for the S4 binding pocket in the active site of GHP3, showed inhibitory activity toward GHP3 protease, using two recombinant peptide substrates. The result suggests that antibodies to chimeric proteins carrying copies of a surface loop of the enzyme active center can be an effective tool for suppressing protease activity. This opens the way to develop more sophisticated approaches to the usage of antibodies in various recombinant forms, that is, scFv fragments. Future work would involve the production of monoclonal or recombinant single‐chain antibodies to similar simple chimeric proteins which could be used in food technologies for control of unwanted protease activity. Antibodies expressed in vitro could be added to flour before dough making, to inhibit protease action. A more radical approach for the future might be the expression of specific antibodies in plants during seed development. DNA encoding scFv antibodies capable of inhibiting pest proteases could be used for the design of novel, safe‐for‐human wheat forms resistant to pest damage and/or gluten resistant to hydrolysis by damaging proteases.

## CONFLICT OF INTEREST

The abovenamed authors of the submitted manuscript confirm no conflicts of interest.

## ETHICAL APPROVAL

This study does not involve any human testing. This study involved animal testing and was approved by the Institutional Review Board of the All‐Russian Research Institute of Plant Protection.
